# REJECTION OF CARE IN PERSONS WITH DEMENTIA

**DOI:** 10.1093/geroni/igz038.653

**Published:** 2019-11-08

**Authors:** Natalie G Regier, Scott Choi, Laura N Gitlin

**Affiliations:** 1 Johns Hopkins University, Baltimore, Maryland, United States; 2 Towson University Department of Nursing, Towson, Maryland, United States; 3 Drexel College of Nursing and Health Professions, Philadelphia, Pennsylvania, United States

## Abstract

Most individuals with dementia develop significant behavioral problems, also known as neuropsychiatric symptoms (NPS). One problem that continues to plague measurement of NPS is inconsistency of terminology used to describe NPS. For example, in the Neuropsychiatric Inventory-Clinician Rating Scale (NPI-C), a gold standard for measuring NPS, rejection of care (rejection) is not differentiated from agitation or aggression. Rather, behaviors indicative of rejection are categorized as agitation. Using data from 250 persons with dementia who participated in the Dementia Behavior Study, principle components analysis of the NPI-C domain of Agitation identified four behavioral clusters: 1=rejection of care, 2=restlessness, 3=exiting behaviors, and 4=hiding/hoarding. Rejection was associated with a more distant relationship with the caregiver, lower cognitive status, and more negative caregiver communication style. Rejection was predictive of higher levels of caregiver burden. Findings support the argument that rejection is a clinically distinct NPS, and likely requires different nonpharmacological management than agitation.

